# Minding the Minds: A Primer on Cognitive Challenge for Marine Mammals in Human Care

**DOI:** 10.3390/ani14060949

**Published:** 2024-03-19

**Authors:** Kelly Jaakkola

**Affiliations:** Dolphin Research Center, Grassy Key, FL 33050, USA; kelly@dolphins.org

**Keywords:** cognitive enrichment, cognitive welfare, animal welfare, cognition, cetaceans, pinnipeds

## Abstract

**Simple Summary:**

Over the past several decades, the attention paid to the physical needs of marine mammals in zoos and aquariums has led to steady improvements in their physical well-being. Up until now, however, we haven’t paid as much attention to their cognitive needs. Studies have suggested that providing animals with puzzles and games that engage their thinking skills can improve their welfare. To help marine mammal managers and trainers achieve this for the animals in their care, this paper discusses different ways of creating such puzzles and thinking games, along with the pros and cons of each.

**Abstract:**

The past several decades have witnessed significant improvement in the physical welfare of marine mammals in zoos and aquariums. Over that same time period, research has revealed complex cognitive abilities in at least some of these species, yet there has been comparatively little attention paid to addressing their cognitive welfare per se. Studies primarily conducted with terrestrial animals have suggested that providing appropriate cognitive challenges in managed care settings can improve animal well-being. As a step toward facilitating this practice with marine mammals, this paper discusses factors relevant for creating appropriate cognitive challenges, outlines the three major categories of cognitive challenge that have been utilized with marine mammals, along with the logistical pros and cons of each, and calls on organizations that care for marine mammals to cultivate a bias for action with respect to providing cognitive care.

## 1. Introduction

Marine mammals have lived in zoological facilities such as zoos, aquaria, and marine mammal parks for more than the past century. During that time, studies have suggested that at least some of these species possess complex cognitive and communicative abilities, including imitation [1,2,3,4]; metacognition [5]; mirror self-recognition [6]; vocally-coordinated cooperation [7]; the ability to learn symbols and basic syntax [8,9,10]; and decades-long memory [11,12]. Over this same time period, thanks to advances in veterinary knowledge and husbandry care, there has been significant and ongoing improvement in the physical welfare of these animals, as evidenced by increases in their health, longevity, and breeding success [13,14,15,16,17,18,19,20]. To maximize their overall well-being, however, their cognitive needs must also be considered.

When considering any animal’s cognitive abilities, it is useful to keep in mind that those abilities originally evolved to solve problems in the service of meeting important life challenges [21], such as how to find and procure food; how to hide from, evade, or drive off predators; how to find or create shelter; how to avoid and deal with injuries; and how to manipulate, cooperate, and compete with social conspecifics. The successful navigation of these important, high-stakes challenges can mean the difference between life and death for animals and their offspring.

For animals who live in zoological facilities, however, these cognitive challenges are typically not as numerous or as difficult. Food and shelter are provided, there is no need to evade or escape predators, veterinary care is provided, and although most animals in these environments will have social relationships with conspecifics, their social landscape is typically relatively simple and highly managed. In other words, in contrast to the situation in the wild, these animals live in highly predictable and structured environments where their primary physical needs—food, shelter, and safety—are generally met. 

On the one hand, this can clearly be viewed as positive, as these animals’ life-dependent challenges have been solved. Indeed, providing animals with adequate food, shelter, and safety is universally considered fundamental to ensuring their positive welfare (e.g., [22,23,24,25,26,27]). That said, however, there is also a potential question about how this might impact their psychological well-being. That is, if an animal’s mind evolved to solve problems, and those problems disappear, what is that mind supposed to do? And how does this impact the animal’s welfare?

## 2. Beyond Physical Welfare

Traditionally speaking, this is where environmental enrichment comes into play. For the past several decades, zoological facilities have recognized the importance of providing animals with environmental enrichment, the goal of which is to “provide the environmental stimuli necessary for optimal psychological and physiological well-being” [28] (p. 1). Extensive scientific evidence has demonstrated that such enrichment can result in numerous behavioral effects indicative of increased positive welfare, such as increased activity, greater behavioral diversity, expanded habitat use, a reduction in fear and reactivity to stressors, and a reduction in stereotypic behavior (e.g., [29,30,31,32,33,34]). Accordingly, environmental enrichment has become an important and standard husbandry practice at zoos and aquaria throughout the world [22,23,24,27,35].

By far the simplest and most common enrichment provided for both terrestrial and aquatic animals in zoological facilities are “toys”—that is, physical objects that the animals can manipulate and move around [36,37,38,39]. For marine mammals specifically, these usually take the form of floating plastic or rubber objects such as balls, hoops, and buoys [37,38,39]. Studies have shown positive benefits of this type of enrichment [31,33,40]. However, simply introducing objects into an animal’s enclosure may not produce consistent and long-term benefits, as animals may habituate to such toys over time [34,41].

Beyond toys, it has been suggested that providing animals with choice and control in their environment can result in further indications of improved welfare (e.g., [42,43,44]). Studies have shown, for example, that when animals can choose whether to be inside or outside [45,46], control the timing of when they receive food or treats [47,48,49,50], or control when music or a light is on or off [47,50,51], this can result in less agitation, a reduction in stereotypic behaviors, calmer activity patterns, lower cortisol levels, more exploratory behavior, and more social play. And when marine mammals are given increased choices within training sessions, such as ways to explicitly choose reward types, behaviors, partners, or even to end the session, this has led to increased interest and participation [52,53].

The question up for consideration in the current paper, however, is whether even this is enough to provide animals with optimal psychological welfare. As noted earlier, minds evolved to meet cognitive challenges. While enrichment in the form of toys, choice, and control certainly provide animals with increased behavioral opportunities and are demonstrably important for welfare, none of these are necessarily challenging. So a reasonable question is whether providing additional cognitive challenge in these structured environments might further enhance animal well-being.

## 3. Benefits of Cognitive Challenge

There are two types of evidence that suggest that adding cognitive challenges to an animal’s environment in managed care settings can in fact enhance animal well-being. First, studies have shown that cognitive challenges themselves can be intrinsically rewarding for a variety of animals. This is demonstrated by the fact that they will (a) choose to work on cognitive puzzles or tasks that have no external reward associated with them [54,55,56,57]; (b) choose to perform a cognitive task to get a reward rather than simply taking an identical reward that is freely available [58,59]; (c) occasionally ignore or discard rewards obtained from doing cognitive puzzles in favor of continuing to work on additional cognitive puzzles [60]; and (d) approach a feeding session with a cognitive task more quickly than a feeding session without such a task [61]. Each of these suggests that there is something intrinsically reinforcing about performing such puzzles and tasks that goes beyond associated external reinforcers.

A second reason to believe that cognitive challenges can enhance animal well-being comes from studies showing that engaging in cognitive challenges has been associated with positive welfare indicators, including increases in movement or activity [62,63,64,65]; decreases in stereotypies [62,63,66,67,68]; decreases in self-directed behaviors such as scratching or feather-plucking that are commonly seen as stress-induced [69,70]; decreases in physiological indicators of stress such as salivary cortisol [65,71]; increases in behavioral diversity and exploratory behavior [62,72]; and the presence of positive affect and excitement behaviors [73,74]. 

That said, it is important to note that cognitive challenges may not always correlate with only positive welfare indicators. For example, studies with chimpanzees [75,76,77,78], bonobos [79], gorillas [75], and mandrills [80] have found that when a cognitive task significantly increased in difficulty or when the animals made errors, there was an increase in their negative emotional expressions and/or self-directed behaviors, indicative of stress or frustration. However, we need to take care not to confuse temporary stress and frustration with longer-term welfare. Each of these studies specifically measured short-term emotional expression, and not longer term effects. It is not at all clear that such temporarily negative emotions should persist or result in any negative outcome. All of us have, at one time or another, felt the temporary frustration of struggling with a difficult task give way to subsequent elation upon solving that same task. Indeed, in their article “The Challenge of Challenge”, Meehan and Mench [81] explicitly discuss temporary frustration as part of the process, defining appropriate challenge (whether cognitive or behavioral) as “problems that may elicit frustration, but are potentially solvable or escapable through the application of cognitive and behavioral skills” (p. 248). This also highlights a crucial difference between solvable and unsolvable problems. Whereas solvable problems encourage problem-solving behavior and overcoming temporary frustration, likely leading to positive welfare outcomes, unsolvable problems prolong unresolved frustration, likely leading to negative welfare outcomes [81]. This means that for enrichment purposes, it is important to aim not just for cognitive challenge, but for appropriate cognitive challenge—which of course begs the question: “What makes an appropriate cognitive challenge?”.

## 4. Defining and Providing Appropriate Cognitive Challenge

In practice, the implementation of cognitively challenging enrichment in zoos and aquariums faces a number of logistical hurdles, perhaps the first being that there is wide disagreement about what it is. A 2021 survey of staff members from zoological facilities around the world found that while there was universal agreement that it is “very important” to provide cognitive enrichment, there was very little consensus on what types of experiences this actually entailed [82]. 

In scientific studies, the term cognitive enrichment has been used to refer to a variety of tasks and situations, depending on species and context, including the following: formal education and/or job complexity (in humans) [83]; a puzzle apparatus that animals figure out how to manipulate to release a toy or food [84,85,86,87,88,89,90,91]; positive reinforcement training mediated by humans (e.g., “clicker training”) [92,93]; automated positive reinforcement training (e.g., an automatic feeding station at which an animal is reinforced for performing a specific task) [63,94,95,96]; interactive computer or touchscreen tasks (e.g., searching for and touching a virtual target; navigating a virtual maze); and cognitive research or other tasks involving learning and/or problem solving (e.g., [37,91,97,98]). 

To characterize and better standardize the meaning, Clark [99] proposed to define cognitive enrichment as “a task (or tasks) whose use (1) engages evolved cognitive skills by providing opportunities to solve problems and control some aspect of the environment, and (2) is correlated to one or more validated measures of wellbeing”. This definition is useful for providing a broad scientific framework, and has been utilized in multiple studies researching the welfare effects of cognitive enrichment (e.g., [72,84,86,87,88,89,90,97]). However, for the practical purposes of figuring out and providing cognitive care, there are two issues for which additional clarification and discussion may be useful.

First, there is the question of what is meant by engaging cognitive skills. Cognition, broadly defined, refers to the ways in which animals take in information through their senses, process and store that information, and decide how to behave on the basis of that information [21,100]. As such, almost all behaviors—from seeing or hearing something to doing something—engage cognitive skills at some level. Consider the simple act of eating food from a bowl. Without further specification, one could theoretically argue that this behavior meets the first part of Clark’s definition, as it engages cognitive skills (i.e., perceiving the food, recognizing it as food, and deciding to eat it), provides an opportunity to solve a problem (i.e., how to gain sustenance), and controls some aspect of the environment (i.e., how much food to eat and how much to leave). However, most people would not consider this to be an example of cognitive enrichment [82]. The problem here is that the term “engaging” is too broad. To remedy this, I would suggest modifying Clark’s definition to replace “engaging” with the implied but not explicitly stated goal of “challenging” the animal cognitively. (See also [101].) 

The second issue centers on the tension between theoretical ideal and practical utility. Specifically, the second part of Clark’s definition requires that the cognitive enrichment task be correlated to at least one validated measure of well-being. This is a wonderful theoretical ideal. After all, something is only enriching if it actually enriches. However, for the practical purposes of providing important cognitive stimulation to the animals we care for, the problem is that there have been few (if any) cognitive tasks that have as yet been correlated to validated measures of well-being for the vast majority of species. If strictly applied, this requirement could thus lead to a practical paralysis and subsequent reduction in cognitive enrichment opportunities, when it is already among the least applied types of enrichment. Between 1985 and 2004, cognitive enrichment was included in less than 4% of enrichment studies for both zoo and laboratory animals, and in 0% for farm animals [36]. And while there have been considerably more studies on cognitive enrichment since that time, it still appears to be among the least used enrichment types [39,101]. 

Given this relative shortage of explicit attention to animals’ cognitive needs in the enrichment literature, I would suggest that the optimal solution is not to wait until a potential form of cognitive enrichment has been scientifically validated for a particular species before attempting it as a cognitive challenge. Three decades ago, Keeling et al. [102] made a similar argument with respect to implementing behavioral enrichment programs for primates, noting that “the absence of complete data should not necessarily subvert immediate action. Because overintellectualizing a problem can lead to paralysis, we must be predisposed to taking action, even though that action may be based on less information than we would like” (p. 58). In a similar way, I would argue that any organization that cares for animals should also cultivate such a bias for action with respect to providing cognitive challenge opportunities. For progress to occur, we must empower animal caretakers to attempt new and different types of cognitive enrichment. Of course, following best practices for any enrichment [35,103], this should ideally be coupled with the practice of documenting the effects of that cognitive challenge, and then evaluating and re-adjusting as necessary. Over time, this iterative process of innovation and evaluation should lead to progress, both in taking care of the cognitive needs of individual animals, and—if the results of such innovation and evaluation are published—advancing the science so that we can eventually meet the theoretical ideal encapsulated in the second part of Clark’s [99] definition.

As a step toward furthering this endeavor, the rest of this article will discuss factors to consider in creating appropriate cognitive challenges, and then outline the three major classes of cognitive challenge that have been utilized with marine mammals specifically, along with the logistical pros and cons of each.

## 5. Creating Appropriate Cognitive Challenge(s)

### 5.1. Species Differences

As noted earlier, cognition can be thought of as the ways in which animals take in, process, store, and act on information [21,100]. To that end, all animals are equipped with a set of perceptual abilities that bring information into the mind, cognitive abilities that process and store that information, and behavioral abilities that allow them to act on that processed information. It is important to recognize, however, that these specific perceptual, cognitive, and behavioral abilities are naturally going to be different across different animal species, given that their bodies and minds have evolved to occupy different environmental niches [21]. Because of this, it is important to design any cognitive challenge we provide an animal to fit with their species’ particular perceptual, cognitive, and behavioral capabilities. Just like we would not expect every animal species to need or to do well with the same physical challenges, we similarly should not expect them to need or to do well with the same cognitive challenges.

One ideal place to begin designing cognitive challenges for any particular species is to look at the kinds of problems that they typically solve in the wild, under the assumption that their cognitive abilities presumably evolved to meet those types of challenges [21]. However, given that we may never know every natural challenge that a given species faces, or every cognitive component involved in that challenge, or be able to reproduce each challenge adequately in a managed care setting, I would also argue that there is great need for more cognitive research to be conducted with every animal species that is kept in managed care environments—whether in zoos, aquariums, laboratories, farms, or human homes. The better we understand the cognition of any particular species, the better we will be able to match the cognitive challenges we design for them with their actual cognitive abilities.

### 5.2. Individual Differences

In addition to species differences, there are also going to be individual differences within species that can influence the effectiveness of any particular cognitive challenge. Studies have shown that these differences can arise at multiple levels, including (a) differences in how interested and motivated specific individuals are to engage with a particular cognitive task in the first place (e.g., [57,86,104,105,106]); (b) differences in the cognitive abilities of specific individuals to solve a particular task (e.g., [107,108,109]); and (c) differences in the extent to which specific individuals show evidence of stress when they have difficulty with a particular cognitive task [76].

Fortunately, animal caretakers and trainers typically have a great deal of knowledge about the individual personalities of the animals they care for. For optimal welfare benefits, I would encourage zoos, aquariums, and other animal facilities to utilize this knowledge in order to adjust, modify, and/or invent challenges with these individual differences in mind.

### 5.3. Adjusting the Challenge Level

Finally, even when working within an appropriate cognitive challenge for a given individual of a given species, the appropriate level of that challenge is going to change over time [110]. Across both areas and species, studies have found that a moderate degree of challenge is most likely to result in the most engagement [111]. Too easy is boring; too difficult is frustrating. For example, with respect to play behavior, animals seem to be most stimulated by environments that are moderately tricky in some way, such as sloped terrains, flexible branches, and fresh snow—topographies that are challenging to navigate, but are also unlikely to result in serious injury [111]. Similarly, when learning operant tasks like pressing a lever to obtain a reward, animals—perhaps counterintuitively—are most engaged when the pay-off for their effort is moderately uncertain. That is, the payoff is not guaranteed every time, but it is also not extremely unlikely [112]. And within human gameplay, it has been found that players are the most engaged when games both match and challenge their level of skill (e.g., [110,113,114]), that is, when the game is difficult enough to challenge their abilities, but not so difficult that winning is nearly impossible.

Given this importance of balancing skill level and challenge, we should expect that as an animal’s experience with a specific cognitive task leads to changes in their skill level, the level of optimal challenge for that animal will also necessarily change. Therefore, if we want to keep our cognitive challenges challenging, they will need to change as well.

## 6. Providing Cognitive Challenges to Marine Mammals

This final section of this paper describes the major categories of cognitive challenge that have been used with marine mammals, and specifically considers a few key factors that may impact their practicality and effectiveness as cognitive enrichment in real-world settings. These factors include (a) how difficult the equipment is to create and maintain, especially in a marine environment; (b) how difficult it is to change the level of challenge to adapt to an animal’s increasing skill levels; and (c) whether it allows for the animals to explore the challenge independently. Given that previous studies have found caretaker time to be the most cited practical factor limiting the provision of enrichment to animals in zoos [82,115], this question of whether a caretaker needs to be physically present during the animal’s use of the challenge may be an important consideration.

### 6.1. Physical Puzzles

The first type of cognitive challenge that is used with animals in zoos and other managed care settings are physical puzzles [34,81]. For the purpose of providing cognitive challenge as discussed here, such puzzles are specifically defined as physical apparatuses for which the animal needs to figure out how to manipulate it to produce some result. This result is most commonly the release of food or a toy, but could also theoretically include any type of overt effect such as interesting movements or sounds. Excluded from this definition are the common practices of food scattering and simple food hiding, in which food items are either mixed with inedible substrates such as wood chips so that the animal has to sift through the substrate to find the edible pieces, or are placed inside or under containers distributed throughout the habitat. Because these practices make food more difficult to access, they are likely effective for increasing physical activity and decreasing idle time, both of which may be beneficial for the animal’s welfare. However, they do not address the goal of cognitively challenging the animal that is under consideration here [81,101].

Some examples of physical puzzles that have been used with marine mammals include an underwater maze that the animals have to maneuver a ball through [86]; a transparent box that requires multiple weights to be deposited to release fish [116,117]; a box that requires two animals to simultaneously pull on different ropes in order to release the toys inside [118]; and a toy submarine that can be activated and steered by using different frequencies of whistles [119]. Note that the key component here is that the animal has something to figure out. Once the animal thoroughly understands and is proficient at how to manipulate the apparatus to get the effect, it ceases to be a puzzle. At this point, it may still provide entertainment and physical enrichment, but without further changes, that particular apparatus can no longer be considered cognitively challenging. 

In terms of practical logistics, perhaps the most important consideration for creating such puzzle apparatuses for marine mammals is the fact that most marine mammals do not have hands or paws with which to manipulate things. This means that instead of constructing an apparatus consisting of small parts for fine motor movements, puzzle apparatuses for marine mammals must be optimized for big movements from big animals. For spatially based puzzles, like Clark et al.’s [86] ball maze constructed out of PVC pipes for dolphins, this means that the apparatus itself will need to be quite large. For smaller options, one could design a puzzle in which the challenge is not spatially based per se, but rather is based on either some other physical characteristic, such as inserting a correctly shaped object to achieve the desired effect, or a socio-cognitive ability such as coordinating action with a social partner.

To increase the difficulty in a physical puzzle, one must be able to change the specific rule or action pattern required to solve the puzzle. This requires either making a physical change in the apparatus, such as rearranging the PVC pipes in a maze to create a different solution path, or replacing the apparatus altogether. The first goal should be to create variability, such that the animal cannot simply re-solve the puzzle by a rote action pattern, but must again actively engage problem solving skills. A more advanced goal would be to make the solution itself more difficult rather than just different, which might be accomplished by increasing the number of steps required to reach the solution, or increasing the difficulty of the steps themselves (such as requiring more precise discriminations in a shape discrimination task). 

As with any type of enrichment, any puzzle device introduced into an animal’s environment should initially be evaluated for safety concerns, and monitored periodically for potential social issues that could arise between animals over its use. Barring such problems, however, a positive feature of physical puzzles is that they generally allow the animals to explore and use them independently, and are thus not limited to those times in which a trainer or caretaker is physically present.

### 6.2. Virtual Puzzles

A second type of cognitive challenge that is becoming increasingly prevalent for terrestrial animals are virtual puzzles or games presented on a computer interface (see reviews in, e.g., [120,121]). Although thus far many of these animal computer systems have been implemented for the purpose of conducting cognitive research rather than for providing enrichment, there is evidence that such cognitive tasks do in fact provide enrichment [120,121,122], and such tools could easily lend themselves to presenting appropriate cognitive challenges even outside of a specific research study. As with physical puzzles, the key for virtual puzzles is that they must include something for the animal to figure out. For example, simply watching videos on these screens would not count as a cognitive challenge, but doing the initial cognitive work of learning how to interface with the system and select particular videos would. Some examples of virtual puzzles that have been used with marine mammals to date have included the following: learning how to maneuver a cursor to contact a target on the screen [123], tracking and touching moving images [124], visual match-to-sample [125], learning to match visual stimuli to associated auditory stimuli [56], and using echolocation to draw [126].

Logistically speaking, there are two major hurdles that must be overcome in order to provide virtual puzzles to marine mammals. The first is finding a way to adapt technology that has been designed for humans so that it can be used by animals with very different body structures and capabilities [127]. The second is the marine environment itself, since computers are typically not designed for underwater use, and will cease to function if they get wet. The few successful attempts at providing computers to marine mammals so far have addressed these problems in one of three ways. All of the groups working with dolphins have placed the computer outside of an underwater window, and then either projected infrared light beams in front of the window, allowing dolphins to use their rostrums to interact with the computer like a touch screen [56,128]; or utilized an array of hydrophones in front of the window that allowed the dolphins to use their echolocation like a computer mouse [126]. The group working with sea lions placed the computer and its controls on land, and used buttons that the sea lion could press with their snout to move a cursor up, down, left, or right [123,125].

Unfortunately, the cost of designing, creating, and maintaining such devices can be quite high, both in terms of funding and expertise. Indeed, in a survey assessing logistical issues with providing computers to apes in zoological facilities, respondents specifically cited cost, the lack of expertise in building devices, and the lack of technological assistance as limiting factors [127]. And realistically, the difficulty of these challenges are only intensified when dealing with an aquatic environment and animals with body shapes and behavioral capabilities so different from humans.

If one can meet these challenges of funding and technological expertise, however, then once a computer interface is in place, the process of adapting the virtual puzzles to match increasing levels of expertise should be relatively straightforward. Unlike physical puzzles that would require a physical change in the apparatus to change anything about the puzzle, virtual puzzles can be changed by updating the computer code, or by writing the initial code so that the game automatically adapts to skill level based on the user’s performance. Such dynamic difficulty adjustment (DDA) has become an increasingly popular feature in recent years for educational and entertainment-based computer games designed for humans [129,130].

In theory, a computer interface for marine mammals might be set up in such a way that it would not require caretaker or trainer oversight every time the animals use the system [120]. This would depend on both safety and training considerations. If any breakable components of the system are within the animals’ reach, or if the use of the virtual games is mediated by trainer reward (e.g., [123,125]), then caretaker oversight is required. But if breakable components are protected from direct contact and the puzzles are designed to be utilized independently (e.g., [56,124]), then the computer could theoretically be used even when a caretaker or trainer is not physically present.

### 6.3. Positive Reinforcement Training

Finally, the third and most common method by far of presenting cognitive challenges to marine mammals is through positive reinforcement training. In modern zoological facilities, such training typically occurs several times each day, and has been shown to have enriching effects on animal welfare (e.g., [131,132,133,134]). Within training sessions, different tasks are likely to provide more or less cognitive challenge. For example, while repeatedly performing an already learned physical behavior provides physical exercise, which can benefit the animal’s welfare, it would not qualify as a cognitive challenge per se. However, learning a new physical behavior would.

In addition to new physical behaviors, training can also provide cognitive challenges by teaching the animals games and puzzles that are specifically designed to exercise cognitive skills such as memory, creativity, problem-solving, and so forth. One form of this is through “concept behaviors”, in which a given signal from the trainer asks the animal not to perform a specific physical behavior, but rather to apply an abstract rule to the situation to figure out what behavior to do [135,136]. For example, “repeat” asks the animals remember the behavior they just did, and do it again; “innovate” asks them to remember the behavior(s) they just did, and do something different (which can be extended into a chain of different behaviors by giving the signal multiple times in succession); and “combo” asks them to combine two or more behaviors together that were not trained together, such as spinning while vocalizing, or diving while wiggling their flippers.

A second source of thinking games comes from research tasks that were originally developed to study marine mammal cognition, and are then adapted for use as cognitive enrichment [37]. Some examples might include the following: match to sample, in which an animal is asked to select which item is the same as an indicated target item (e.g., [137,138]); imitation, in which one animal is asked to do the same behavior that another animal is doing [3,4,139]; memory for spatial locations, in which an object is hidden and the animal is asked to select where the object is [140]; and feature comparison, in which the animal is presented with two stimulus items and is asked to choose the one that has either more or less of a given feature (such as the one with the greater quantity of dots; or the one that is smaller in size) (e.g., [141,142,143]).

Logistically speaking, using positive reinforcement training for delivering cognitive challenges has a couple of distinct advantages. First, minimal equipment is needed to create the challenge. Almost all of the thinking games discussed here require only what would normally be brought to a training session, plus perhaps some toys and buckets. Second, dynamically adapting the challenge to the animal’s skill level is already an inherent part of the positive reinforcement training process, so no additional resources are required to increase the challenge level. The downside, of course, is that, by definition, training games are trainer-dependent, rather than something that the animals can continually explore on their own. Therefore, these types of cognitive challenges can only be utilized during the parts of the day in which trainers are physically present and actively conducting sessions.

## 7. Conclusions

In conclusion, providing optimal care for marine mammals requires the recognition that animal minds evolved to solve problems. Because ensuring their physical well-being in zoological facilities rightly involves solving most of these problems (e.g., procuring food, shelter, and safety), current practices may in fact be creating an unintentional trade-off between optimal physical and psychological well-being. To address this, studies have suggested that adding appropriate cognitive challenge to an animal’s environment can enhance positive welfare in zoological settings.

In order to do this effectively, more research is needed, both on the basic cognitive abilities of different animal species, and on the welfare effects of providing them with different types and levels of cognitive challenge. To date, the vast majority of this type of research has been conducted with primates [144,145], animals who differ dramatically from marine mammals in environment, body structure, and perceptual and behavioral capabilities. Within marine mammals, there is also a large asymmetry, with the most research on cognition and cognitive welfare being conducted with bottlenose dolphins [37,146]. Given that animal species differ in their cognitive abilities due to different evolutionary histories and ecological niches, it is important to conduct this research with each species in our care to most effectively optimize the cognitive challenges that will ensure their best welfare.

As this research is being conducted, however, we also need to cultivate a bias for action with respect to cognitive care. While the physical well-being of marine mammals in zoological facilities has improved steadily over the past several decades, there has as yet been significantly less explicit attention paid to ensuring their cognitive well-being. And while the recent past has seen improvement in providing environmental enrichment as well as more choice and control, marine mammals possess a number of complex cognitive abilities that in many facilities may not be engaged or challenged on a regular basis. Therefore, in order to continue the trajectory of ongoing improvement in welfare, I would argue that the ideal next step would be to devote more attention, action, and resources to the goal of developing challenges to specifically enhance the cognitive well-being of marine mammals in zoological settings.

## Data Availability

Not applicable.
